# A prospective evaluation of serum kynurenine metabolites and risk of pancreatic cancer

**DOI:** 10.1371/journal.pone.0196465

**Published:** 2018-05-07

**Authors:** Joyce Y. Huang, Lesley M. Butler, Øivind Midttun, Arve Ulvik, Renwei Wang, Aizhen Jin, Yu-Tang Gao, Per M. Ueland, Woon-Puay Koh, Jian-Min Yuan

**Affiliations:** 1 Division of Cancer Control and Population Science, UPMC Hillman Cancer Center, University of Pittsburgh, Pittsburgh, Pennsylvania, United States of America; 2 Department of Epidemiology, Graduate School of Public Health, University of Pittsburgh, Pittsburgh, Pennsylvania, United States of America; 3 Bevital A/S, Bergen, Norway; 4 Department of Clinical Science, University of Bergen, Bergen, Norway; 5 National Registry of Diseases Office, Health Promotion Board, Singapore, Republic of Singapore; 6 Department of Epidemiology, Shanghai Cancer Institute/Shanghai Jiaotong University, Shanghai, China; 7 Department of Clinical Science, University of Bergen, Bergen, Norway; 8 Laboratory of Clinical Biochemistry, Haukeland University Hospital, Bergen, Norway; 9 Duke-NUS Graduate Medical School Singapore, Singapore, Republic of Singapore; 10 Saw Swee Hock School of Public Health, National University of Singapore, Singapore, Republic of Singapore; Centro Nacional de Investigaciones Oncologicas, SPAIN

## Abstract

**Background:**

Serum pyridoxal 5’-phosphate (PLP), the active form of vitamin B_6_, is associated with reduced risk of pancreatic cancer. Data on functional measures of vitamin B_6_ status and risk of pancreatic cancer is lacking.

**Methods:**

A nested case-control study involving 187 incident cases of pancreatic cancer and 362 individually matched controls were conducted within two prospective cohorts to evaluate the associations between kynurenine metabolites in pre-diagnostic serum samples and risk of pancreatic cancer.

**Results:**

Higher serum concentrations of 3-hydroxyanthranilic acid (HAA) and the HAA:3-hydroxykynurenine (HK) ratio (a measure for *in vivo* functional status of PLP) were significantly associated with reduced risk of pancreatic cancer. Compared with the lowest tertile, odds ratios (95% confidence intervals) of pancreatic cancer for the highest tertile was 0.62 (0.39, 1.01) for HAA, and 0.59 (0.35–0.98) for the HAA:HK ratio, after adjustment for potential confounders and serum PLP (both *P*s for trend<0.05). The kynurenine:tryptophan ratio or neopterin was not significantly associated with pancreatic cancer risk.

**Conclusions:**

The inverse association between HAA or the HAA:HK ratio and risk of pancreatic cancer supports the notion that functional status of PLP may be a more important measure than circulating PLP alone for the development of pancreatic cancer.

## Introduction

Pancreatic cancer is among the deadliest malignancies in the world [1]. Few primary prevention strategies are available for pancreatic cancer; only one-third of pancreatic cancer burden is attributed to smoking and obesity, the two established modifiable risk factors of pancreatic cancer [2, 3]. Studies are needed to identify novel risk or protective factors for pancreatic cancer in order to develop strategies for monitoring high-risk populations and reducing risk through primary prevention intervention.

Recently much attention has been paid to the metabolomics biomarkers in relation to the risk assessment or clinical diagnosis of pancreatic cancer [4–7]. Pyridoxal 5’-phosphate (PLP), the active form of vitamin B_6_, plays a significant role in multiple biological mechanisms that can modulate carcinogenesis, including DNA methylation and synthesis, antioxidant defense system, and inflammation [8]. Our group previously reported that dietary intake of vitamin B_6_ and/or PLP level in serum samples collected prior to cancer diagnosis were inversely associated with risk of pancreatic cancer [9, 10].

**Fig 1:**
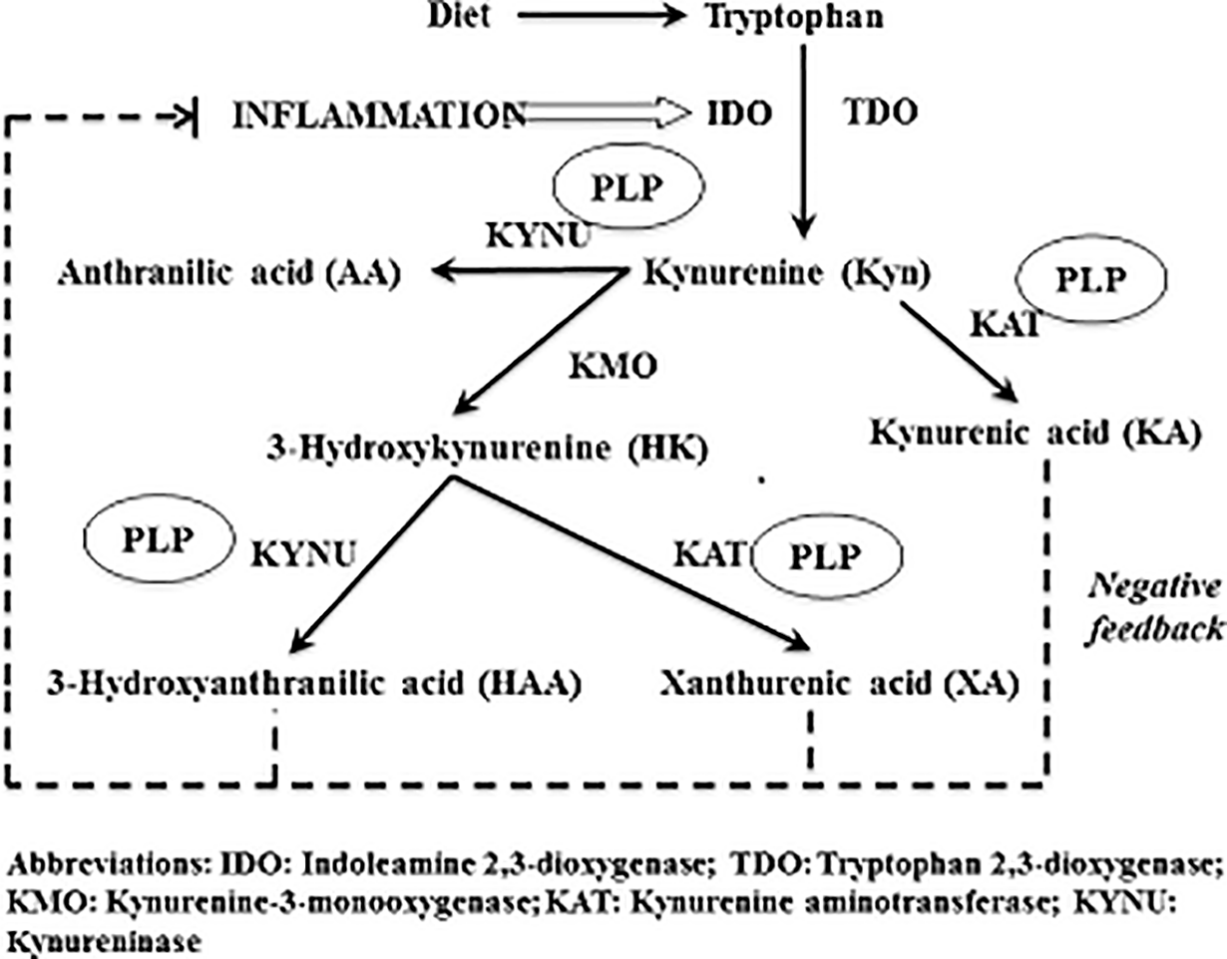
The schematic metabolic pathway of tryptophan and kyurenine.

PLP plays an important role in the kynurenine pathway of the tryptophan metabolism (**Fig 1**). Kynurenine is metabolized to anthranilic acid, xanthurenic acid (XA), kynurenic acid (KA), and 3-hydroxyanthranilic acid (HAA) via PLP-dependent enzymes. Ratios of kynurenine metabolites, such as XA:3-hydroxykynurenine (HK), HAA:HK, and KA:HK have been proposed as indicators of intracellular functional status of PLP [11].

Kynurenine pathway is also involved in inflammation and immune function via depletion of tryptophan and immuno-regulatory effects of downstream metabolites [12]. For example, kynurenine, HK and HAA are redox active compounds, with HAA being a potent antioxidant with anti-inflammatory effects [13]. Ratio of kynurenine to tryptophan (KTR) and neopterin, a metabolite of guanosine triphosphate, are markers of interferon (IFN)-gamma-induced immune activation [14]. Although some studies reported that KTR and neopterin were associated with increased risk of several cancers and all-cause mortality [15–19], their association with risk of pancreatic cancer has not been investigated.

We conducted a case-control study nested within two prospective cohorts to evaluate the association between kynurenine metabolites and their ratios as functional measures of PLP and risk of pancreatic cancer.

## Methods

### Study subjects

Detailed study design, selection of cases and controls, and statistical methods have been published previously [10]. Briefly, the present case-control study included 187 incident pancreatic cancer cases and 362 individually matched controls nested within the Shanghai Cohort Study and the Singapore Chinese Health Study. The Shanghai Cohort Study enrolled 18,244 male residents aged 45 to 64 years in Shanghai, China from 1986 through 1989 [20]. At the time of enrollment, demographic and lifestyle information was collected from study subjects through an in-person interview. Non-fasting blood and urine samples were collected immediately after the completion of interview and stored at -80°C until laboratory analysis. The Singapore Chinese Health Study enrolled 63,257 Chinese men and women aged 45 to 74 years in Singapore from 1993 through 1998 [21]. Information on demographics, height, body weight, history of tobacco and alcohol use, and medical history was collected from study participants through an in-person interview at baseline. Non-fasting blood and urine samples were collected after baseline interview between 1994 and 2005 and stored at -80°C until laboratory analysis. Written informed consent was obtained from all cohort participants. Both cohort studies were approved by the Institutional Review Board of the University of Pittsburgh, the Shanghai Cancer Institute, and the National University of Singapore.

### Case ascertainment and control selection

In the Shanghai cohort, all surviving cohort participants were re-contacted annually. As of the most recent follow-up in 2015, 3.7% of original cohort participants were lost to the follow-up interview and 3.3% declined the continued follow-up interview. The incident cancer cases and deaths among cohort participants were identified through annual re-contacts of surviving study participants or next-of-the-kin for deceased participants, and through record linkage analyses with the databases of the population-based Shanghai Cancer Registry and the Shanghai Municipal Vital Statistics Office. The diagnosis of all incident cancer cases was confirmed via review of medical records. As of December 31^st^ 2009, the cut-off date for the present study, 129 incident cases of primary pancreatic cancer [International Classification of Disease (ICD)-9 code, 157] were identified among participants of the Shanghai cohort.

In the Singapore cohort, less than 1% of original cohort members were lost to follow-up due to their migration out of Singapore. The incident cancer cases and deaths among cohort members of the Singapore cohort were identified through routine record linkage with databases of the Singapore National Birth and Death Registry and National Cancer Registry [22]. As of December 31^st^, 2013, 58 incident cases of primary pancreatic cancer (ICD-Oncology code, C25) were identified among participants of the Singapore cohort who had available serum samples.

For each case, two control subjects were randomly selected among all eligible participants who were free of cancer at the time of cancer diagnosis of the index case within the same cohort. To be consistent with the matching criteria used in previous nested case-control studies in the Shanghai cohort, controls were matched to the index case on date of birth (±2 years), date of biospecimen collection (±1 month), and neighborhood of residence at time of enrollment [20, 23]. In the Singapore cohort, cases and controls were matched on age at baseline interview (± 3 years), date of baseline interview (± 2 years), gender, dialect group (Cantonese, Hokkien), and date of biospecimen collection (±6 months).

### Assessment of serum biomarkers

Serum specimens of cases and their matched controls were processed, aliquoted, shipped in frozen state and assayed together at Bevital A/S (www.bevital.no), Bergen, Norway with blind case-control status. Serum tryptophan, kynurenine, KA, XA, anthranilic acid, HK, HAA, neopterin, PLP, creatinine, and cotinine were measured by liquid chromatography-tandem mass spectrometry (LC-MS/MS) [24, 25]. For quality control purpose, 14 duplicated samples separated in seven batches (two per batch) from pooled serum collected from potential cohort participants at the same time period as the study samples. The within-batch coefficients of variation for the biomarkers ranged between 0.9% and 5.5% (see detail in **S1 Table**).

### Statistical analysis

We first calculated the following ratios of kynurenine metabolites. The KA:HK ratio, XA:HK ratio, and HAA:HK ratio are indicators for functional status of PLP. KTR is an IFN-gamma-induced inflammatory marker [26]. Original values of all biomarkers measured and their ratios were logarithmically transformed to normalize their distributions. The analysis of variance (ANOVA) method was used to evaluate the effect of smoking status, body mass index (BMI, kg/m^2^), and serum cotinine and PLP on serum concentrations of tryptophan and kyurenine metabolites and their ratios with adjustment with age, gender, cohort location, month of blood draw, and year of interview.

Study subjects were divided into tertiles based on the distribution of individual biomarkers among controls pooled from two cohorts because the distributions and tertile cut-off values are comparable among controls of these two cohort for all but tryptophan and neopterin (**S2 Table**). Odds ratios (ORs) and their 95% confidence intervals (CIs) of pancreatic cancer for higher tertiles of the biomarkers relative to the lowest tertile were calculated using conditional logistic regression [27]. Ordinal value (e.g., 1, 2, and 3) for each of the biomarkers was used for linear trend test in the biomarker-pancreatic cancer risk association.

The multivariable conditional logistic regression models included the following risk factors for pancreatic cancer as potential confounders: BMI (<18.5, 18.5-<23, ≥23 kg/m^2^), level of education (no formal schooling, primary school, secondary school and above), smoking status (never smokers, former smokers, current smokers), alcohol consumption (number of drinks per week), history of physician-diagnosed diabetes (no, yes), estimated glomerular filtration rate (eGFR) [28], and cohort location (Shanghai, Singapore). Serum cotinine (continuous) was also included in the multi-variable logistic regression model because cotinine was an objective biomarker for recent exposure to nicotine derived from active and second-hand smoking with a Spearman correlation coefficient being 0.635 between serum cotinine and number of cigarettes per day (*P* <0.0001). In addition, we adjusted for serum PLP in the multivariable logistic regression models to assess if the observed associations between the metabolites of kynurenine and risk of pancreatic cancer was due to different levels of PLP or through a possible pathway other than PLP.

Stratified analyses were performed by cohort location (Shanghai, Singapore) and PLP deficiency status (<20, ≥ 20 nmol/L). Potential effect modification on pancreatic cancer risk was assessed by including an interaction term between a biomarker and cohort location or PLP deficiency status in the regression models.

Sensitivity analysis was conducted to examine the potential effect of disease progression on the observed biomarker-disease risk association with the exclusion of cases diagnosed within first several years after blood draw.

Statistical analyses were carried out using SAS software version 9.3 (SAS Institute, Cary, NC). All *P* values reported are two-sided, and those that were less than 0.05 were considered to be statistically significant.

## Results

The mean (standard deviation) age at blood draw for study participants of the Shanghai and Singapore cohorts was 56.4 (5.5) and 64.4 (7.3) years, respectively. The average (range) time interval between blood draw and cancer diagnosis was 12.5 years (3 months to 23.2 years) for cases of the Shanghai cohort, and 6.8 years (5 months to 13.0 years) for cases of the Singapore cohort.

The characteristics of pancreatic cancer cases and matched controls for the Shanghai and Singapore cohorts are presented separately in **Table 1**. In the Shanghai cohort, pancreatic cancer cases were more likely to be current smokers at baseline and had higher levels of serum cotinine, and lower concentrations of serum PLP and neopterin than control subjects (all P values < 0.05). In the Singapore cohort, the concentration of HAA was higher in control subjects than in cases (P value <0.05) while the difference in serum concentrations of other kynurenine metabolites or their ratios was not statistically significant (**Table 2**).

**Table 1:** Baseline demographic characteristics and lifestyle factors in pancreatic cancer cases and control subjects, the Shanghai Cohort Study and the Singapore Chinese Health Study.

	Shanghai cohort			Singapore cohort		
Characteristic or lifestyle factors	Controls	Cases	P^a^	Controls	Cases	P^a^
N	258	129		104	58	
Age at interview, mean (SD), years	56.4 (5.5)	56.5 (5.5)	0.74	57.1 (7.2)	57.9 (7.5)	0.51
Age at blood draw, mean (SD), years	56.4 (5.5)	56.5 (5.5)	0.74	64.0 (7.1)	64.9 (7.6)	0.47
BMI, mean (SD), kg/m^2^	21.9 (2.8)	22.5 (3.0)	0.08	23.1 (3.2)	23.2 (3.6)	0.79
Female (%)	0	0		39.4	39.7	0.98
Education level (%)			0.36			0.42
No formal schooling	5.0	2.3		20.2	12.1	
Primary school	28.7	26.4		43.3	48.3	
Secondary school or above	66.3	71.3		36.5	39.7	
Smoking status (%)			0.003			0.87
Never	43.8	27.1		60.6	58.6	
Former	6.2	4.7		22.1	20.7	
Current	50.0	68.2		17.3	20.7	
Alcohol intake, drinks/week (%)			0.74			0.61
0	56.6	54.3		82.7	87.9	
<7	11.2	14.0		10.6	8.6	
≥7	32.2	31.8		6.7	3.5	
Diabetes (%)			0.52			0.88
No	98.5	99.2		90.4	89.7	
Yes	1.55	0.78		9.6	10.3	
Weekly multivitamin use (%)			---			0.69
No	---	---		89.4	91.4	
Yes	---	---		10.6	8.62	

^a^ 2-sided P values were based on the Student *t* test for difference in means or Chi-square test for difference in percentage (%) between cases and controls within each cohort.

**Table 2:** Median (5^th^– 95^th^ percentile) values of serum biomarkers of tryptophan, kynurenines and neopterin in pancreatic cancer cases and control subjects, the Shanghai Cohort Study and the Singapore Chinese Health Study.

	Shanghai cohort			Singapore cohort		
Biomarker^a^	Controls	Cases	P^b^	Controls	Cases	P^b^
PLP, nmol/L	25.7 (10.0–91.7)	21.7 (8.94–60.0)	0.01	58.1 (20.8–563.0)	50.6 (23.8–465.0)	0.29
Tryptophan, μmol/L	76.5 (57.9–100.5)	74.4 (60.0–105.7)	0.71	69.3 (54.3–95.6)	70.3 (46.5–95.3)	0.67
Kynurenine, μmol/L	1.54 (1.15–2.10)	1.54 (1.14–2.01)	0.40	1.62 (1.12–2.38)	1.66 (1.02–2.19)	0.85
Anthranilic acid, nmol/L	17.7 (10.8–47.7)	18.5 (10.6–89.8)	0.33	21.8 (12.6–55.8)	21.0 (13.1–54.3)	0.59
KA, nmol/L	55.3 (28.2–107.0)	51.6 (22.8–101.0)	0.49	57.7 (28.1–130.0)	52.1 (31.0–156.0)	0.39
HK, nmol/L^c^	45.7 (28.1–80.2)	44.3 (25.2–86.4)	0.72	47.8 (29.8–74.1)	44.2 (27.0–70.7)	0.22
XA, nmol/L	17.7 (8.4–33.1)	17.4 (6.36–39.1)	0.98	17.2 (8.28–32.4)	14.6 (8.74–30.3)	0.09
HAA, nmol/L^c^	37.0 (16.6–66.3)	35.6 (15.9–68.7)	0.16	42.7 (25.2–69.9)	37.6 (23.8–76.5)	0.046
KA:HK ratio	1.16 (0.602–2.13)	1.13 (0.649–2.11)	0.57	1.19 (0.736–2.40)	1.18 (0.741–2.36)	0.66
XA:HK ratio	0.389 (0.194–0.640)	0.391 (0.183–0.753)	0.93	0.363 (0.228–0.591)	0.345 (0.167–0.623)	0.31
HAA:HK ratio	0.825 (0.389–1.31)	0.756 (0.313–1.49)	0.12	0.940 (0.544–1.51)	0.856 (0.457–1.49)	0.39
KTR (x 100)	2.04 (1.52–2.92)	2.02 (1.49–2.78)	0.36	2.30 (1.57–3.54)	2.24 (1.63–3.57)	0.88
Neopterin, nmol/L^c^	14.3 (8.77–24.6)	13.2 (12.8–14.2)	0.04	24.7 (16.0–39.0)	26.6 (16.1–42.2)	0.33
Cotinine, nmol/L	87.5 (1.51–1620.0)	518.0 (1.79–1500.0)	0.005	1.60 (0–848.0)	2.33 (0–1280.0)	0.28
eGFR (mL/min/1.73m^2^)	92.7 (65.7–106.5)	93.3 (7.83–23.3)	0.33	77.2 (45.6–99.8)	76.8 (44.2–106.9)	0.81

^a^ Abbreviations: eGFR, estimated glomerular filtration rate; HAA, 3-hydroxyanthranilic acid; HK, 3-hydroxykynurenine; KA, kynurenic acid; KTR, kynurenine:tryptophan ratio; PLP, pyridoxal 5’-phosphate; XA, xanthurenic acid.

^b^ 2-sided P values were based on the Mann-Whitney test.

^c^ 13 cases and 4 controls were excluded from the present analysis due to missing values for HK, HAA, and neopterin from the Shanghai cohort

Current smoker had higher level of HK and lower KA:HK ratio, HAA:HK ratio and neopterin whereas serum cotinine level was not associated with any of the tryptophan and kyurenine metabolites or their ratios (**Table 3**). The levels of kynurenine, KA, and the KA:HK ratio increased with increasing BMI. PLP was positively associated with KA, HAA, the KA:HK ratio, the HAA:HK ratio and the XA:HK ratio, but inversely associated with HK. Among kynurenine metabolites and their ratios, serum PLP had highest correlation coefficient with HAA:HK ratio (r = 0.39, P <0.0001), followed by HAA (r = 0.27, P <0.0001) (**S2 Table**). Similarly, neopterin was also significantly correlated with PLP (r = 0.39, P <0.0001).

**Table 3:** Beta coefficients^a^ for tryptophan and kynurenine metabolites derived from linear regression models on smoking status (current or former versus never smokers), cotinine, body mass index (BMI), and pyridoxal 5’-phosphate (PLP) among all controls (N = 362).

	Former smokers	Current smokers	Cotinine (per 100 nmol/L)	BMI (per 5 kg/m^2^)	PLP (per 100 ng/mL)
Tryptophan, μmol/L	0.024	-0.001	<0.001	0.026	0.013
Kynurenine, μmol/L	0.043	0.022	<0.001	0.041^b^	0.004
Anthranilic acid, nmol/L	-0.093	-0.103	-0.010	0.094	0.024
KA, nmol/L	-0.045	-0.040	-0.003	0.103^b^	0.064^c^
HK, nmol/L	0.029	0.082^b^	0.006	0.016	-0.067^c^
XA, nmol/L	-0.014	0.011	-0.002	0.084	0.020
HAA, nmol/L	-0.053	-0.051	<0.001	0.080	0.047^b^
KA:HK ratio	-0.091	-0.115^b^	-0.010	0.096^b^	0.131^c^
XA:HK ratio	-0.058	-0.075	-0.010	0.076	0.086^c^
HAA:HK ratio	-0.081	-0.133^b^	-0.010	0.064	0.114^c^
KTR (x 100)	0.019	0.022	<0.001	0.015	-0.009
Neopterin, nmol/L	-0.014	-0.102^c^	-0.010	0.062	0.012

Abbreviations: HAA, 3-hydroxyanthranilic acid; HK, 3-hydroxykynurenine; KA, kynurenic acid; KTR, kynurenine:tryptophan ratio; XA, xanthurenic acid.

^a^ All beta coefficients presented in the table were adjusted for age, gender, month of blood collection, year of baseline interview, and cohort location (Shanghai, Singapore).

^b^ 2-sided P value <0.05

^c^ 2-sided P-value <0.01

Increasing level of HAA was associated with significantly decreased risk of pancreatic cancer (**Table 4**); the multivariate-adjusted OR for the third tertile was 0.62 (95% CI = 0.39–1.00; P for trend = 0.04). Further adjustment for serum concentration of PLP did not alter the risk estimates. Similarly, the HAA:HK ratio was significantly associated with reduced risk of pancreatic cancer; the multivariable-adjusted OR was 0.59 (95% CI = 0.35–0.98; P for trend = 0.04). We also examined the relationship for continuous values of HAA and HAA:HK ratio with risk of pancreatic cancer. The multivariate-adjusted ORs (95% CIs) of pancreatic cancer for log_2_(HAA) and log_2_(HAA”HK ratio) were 0.70 (0.50–0.99; *P* for linear trend = 0.047) and 0.80 (0.55–1.15, *P* for linear = 0.023), respectively. Additional adjustment for alcohol consumption did not materially change the association between these two biomarkers and risk of pancreatic cancer. There was no statistically significant association between other kynurenine metabolites and risk of pancreatic cancer (**Table 4**).

**Table 4:** Associations of tryptophan, kynurenines, and neopterin with pancreatic cancer risk in both Shanghai and Singapore cohorts combined (187 cases and 362 controls).

		Serum concentration of biomarker in tertile			
Biomarker^a^		1^st^ tertile	2^nd^ tertile	3^rd^ tertile	P-trend
Tryptophan	Controls/Cases	120/66	123/63	119/58	
	OR (95% CI) ^a^	1.00 (ref)	0.88 (0.56–1.37)	0.88 (0.56–1.39)	0.55
	OR (95% CI) ^b^	1.00 (ref)	0.87 (0.54–1.38)	0.87 (0.54–1.39)	0.55
Kynurenine	Controls/Cases	120/76	123/56	119/55	
	OR (95% CI) ^a^	1.00 (ref)	0.70 (0.45–1.1)	0.71 (0.42–1.19)	0.16
	OR (95% CI) ^b^	1.00 (ref)	0.70 (0.45–1.1)	0.71 (0.42–1.19)	0.16
Anthranilic acid	Controls/Cases	117/47	120/77	116/59	
	OR (95% CI) ^a^	1.00 (ref)	2.01 (1.21–3.33)	1.53 (0.88–2.67)	0.16
	OR (95% CI) ^b^	1.00 (ref)	2.01 (1.21–3.33)	1.53 (0.88–2.67)	0.16
KA	Controls/Cases	120/69	123/64	119/54	
	OR (95% CI) ^a^	1.00 (ref)	0.98 (0.61–1.56)	0.83 (0.51–1.34)	0.45
	OR (95% CI) ^b^	1.00 (ref)	0.98 (0.61–1.56)	0.83 (0.51–1.35)	0.46
HK	Controls/Cases	119/68	118/62	116/53	
	OR (95% CI) ^a^	1.00 (ref)	0.91 (0.58–1.43)	0.69 (0.42–1.12)	0.13
	OR (95% CI) ^b^	1.00 (ref)	0.90 (0.57–1.42)	0.68 (0.41–1.11)	0.12
XA	Controls/Cases	120/73	124/58	118/56	
	OR (95% CI) ^a^	1.00 (ref)	0.82 (0.53–1.27)	0.83 (0.53–1.32)	0.41
	OR (95% CI) ^b^	1.00 (ref)	0.82 (0.53–1.27)	0.83 (0.52–1.32)	0.41
HAA	Controls/Cases	117/79	120/52	116/52	
	OR (95% CI) ^a^	1.00 (ref)	0.60 (0.37–0.95)	0.62 (0.39–1.00)	0.04
	OR (95% CI) ^b^	1.00 (ref)	0.60 (0.37–0.95)	0.62 (0.39–1.01)	0.04
KA:HK ratio	Controls/Cases	117/64	120/67	116/52	
	OR (95% CI) ^a^	1.00 (ref)	1.11 (0.68–1.8)	0.94 (0.57–1.53)	0.76
	OR (95% CI) ^b^	1.00 (ref)	1.11 (0.68–1.8)	0.94 (0.57–1.56)	0.79
XA:HK ratio	Controls/Cases	117/64	120/67	116/52	
	OR (95% CI) ^a^	1.00 (ref)	0.54 (0.33–0.9)	0.87 (0.54–1.39)	0.60
	OR (95% CI) ^b^	1.00 (ref)	0.54 (0.33–0.9)	0.87 (0.54–1.4)	0.61
HAA:HK ratio	Controls/Cases	117/77	120/58	116/48	
	OR (95% CI) ^a^	1.00 (ref)	0.64 (0.4–1.02)	0.60 (0.37–0.98)	0.04
	OR (95% CI) ^b^	1.00 (ref)	0.60 (0.37–0.98)	0.59 (0.35–0.98)	0.04
KTR	Controls/Cases	120/65	123/67	119/55	
	OR (95% CI) ^a^	1.00 (ref)	0.99 (0.61–1.59)	0.90 (0.53–1.52)	0.69
	OR (95% CI) ^b^	1.00 (ref)	0.98 (0.61–1.59)	0.90 (0.53–1.52)	0.68
Neopterin^c^	Controls/Cases	118/76	120/50	115/57	
	OR (95% CI) ^a^	1.00 (ref)	0.68 (0.42–1.1)	0.84 (0.51–1.4)	0.50
	OR (95% CI) ^b^	1.00 (ref)	0.68 (0.42–1.1)	0.84 (0.51–1.4)	0.49

^a^ Abbreviations: HAA, 3-hydroxyanthranilic acid; HK, 3-hydroxykynurenine; KA, kynurenic acid; XA, xanthurenic acid.

^a^ Odds ratios were derived from conditional logistic regression models that also included the following covariates: education (no schooling, primary school, secondary school and higher), body mass index (<18.5, 18.5-<23.0, ≥23.0), smoking status (never, former, current), serum cotinine concentrations (nmol/L), alcohol drinking (drinks of alcoholic beverages per week), diabetes status (no, yes), estimated glomerular filtration rate (mL/min/1.73 m^2^), and study location (Shanghai vs Singapore).

^b^ In addition to covariates described above, the conditional logistic regression models included serum pyridoxal 5’-phosphate (nmol/L).

^c^ Cohort-specific tertiles was used for neopterin due to its different distribution between two cohorts.

The associations between kynurenine metabolites and risk of pancreatic cancer were similar in both Shanghai cohort and Singapore cohorts (all *P’s* for heterogeneity between the two cohorts ≥ 0.10) (**S4 and S5 Tables).** When analyses were conducted for subjects with serum higher (≥20 nmol/L) or lower PLP (<20 nmol/L), kynurenine, HK, HAA, and the HAA:HK ratio showed stronger inverse association with risk of pancreatic cancer among individuals with lower PLP than among those with higher PLP. However, the differences in these associations between higher and lower PLP groups were not statistically significant **(S6 Table)**. No association was observed between markers of IFN-gamma-induced immune activation, KTR and neopterin, and risk of pancreatic cancer in all subjects (**Table 4**) or in stratified analysis by PLP deficiency status (**S6 Table**). We also examined and did not find any statistically significant difference in the association between metabolites of tryptophan and kynurenine and pancreatic cancer risk in subgroups of study subjects stratified by gender, BMI, smoking status, or alcohol consumption (data not shown).

In the sensitivity analysis after excluding cases diagnosed within first two years of serum collection (13 cases), the associations for risk of pancreatic cancer with HAA and HAA:HK ratio remained. Compared to the lowest tertiles, the PLP-adjusted multivariable ORs of pancreatic cancer for the highest tertile of HAA and the HAA:HK ratio were 0.58 (95% CI = 0.36–0.94, P for trend = 0.02) and 0.60 (95% CI = 0.36–1.00, P for trend = 0.06), respectively. Further excluding cases diagnosed within first 4 years (32 cases) did not materially change the results. The corresponding ORs were 0.59 (95% CI = 0.36–0.97, P for trend = 0.03) and 0.63 (95% CI = 0.37–1.07, P for trend = 0.08).

## Discussion

In this prospective study using serum samples collected on average of 10.7 years before cancer diagnosis, we observed a statistically significant inverse association between HAA and the HAA:HK ratio, markers of functional status of PLP, and risk of pancreatic cancer. Overall, there was a 40% reduction in risk of pancreatic cancer for individuals at the highest third of HAA or the HAA:HK ratio at baseline.

PLP plays an important role in the kynurenine pathway as the cofactor for kynureninase (KYNU) and kynurenine amino transferase (KAT). KYNU catalyzes the production of anthranilic acid and HAA, while KAT catalyzes the production of KA and XA (**Fig 1**). The ratios of HAA:HK, KA:HK, and XA:HK can be used to reflect functional status of PLP [11]. In the current study, we found that higher HAA:HK ratio, but not the KA:HK ratio or the XA:HK ratio, was significantly associated with reduced risk of pancreatic cancer. The discrepancy in the associations of HAA:HK ratio versus KA:HK ratio and XA:HK ratio may be explained by the fact that KYNU is more sensitive to PLP deficiency than KAT [29]. The current findings of inverse associations between HAA and HAA:HK ratio and risk of pancreatic cancer support the notion that PLP may protect against the development of pancreatic cancer.

We previously reported that higher level of serum PLP was associated with more than 50% lower risk of pancreatic cancer compared with the PLP deficiency (<20 nmol/L) [10]. This inverse relationship was primarily driven by the high proportion of individuals with PLP deficiency status in the Shanghai Cohort Study, which comprised men only and approximately 50% current smokers at baseline blood draw [10]. This inverse association between serum PLP and pancreatic cancer was consistent with the results in the Alpha-Tocopherol, Beta-Carotene Cancer Prevention Study in Finland, which included male smokers only [30]. Two other studies did not find a statistically significant association between serum PLP and pancreatic cancer risk in U.S. and Europe, respectively [31, 32]. The inconsistent results between PLP and pancreatic cancer risk in these previous studies could be attributed to different levels of exposure to vitamin B_6_ and other risk factors such as smoking as well as different measure of PLP.

Additional adjustment for serum concentration of PLP did not change any risk estimates of pancreatic cancer for HAA and the HAA:HK ratio, suggesting that there may exist biological mechanism other than PLP. HAA has strong anti-inflammatory properties. For example, HAA inhibits the production of pro-inflammatory interleukin-17 (IL-17) [33]. IL-17 has been shown to accelerate the progression of precancerous lesions in the pancreas of Kras-mutated mice [34]. In addition, HAA is a precursor of cinnabarinic acid, an endogenous ligand to the aryl hydrocarbon receptor (AhR) [35]. The activation of AhR can lead to cell cycle arrest and growth inhibition of pancreatic cancer cell lines through induction of the cyclin-dependent kinase inhibitor p21 [36]. Therefore, the anti-inflammatory properties of HAA may be a biological mechanism for the observed inverse association between HAA and the HAA:HK ratio and risk of pancreatic cancer in addition to serving as functional markers of PLP status. In fact, the inverse association between HAA or HAA:HK ration and risk of pancreatic cancer was much stronger in individuals with deficient PLP than those with sufficient PLP in the present study. A study with a larger sample size would be required to separate the effect of PLP from other kyurenine metabolites influenced by PLP.

KTR and neopterin are markers of IFN-gamma-induced immune activation. IFN-gamma upregulates the activity of indoleamine 2,3-dioxygenase (IDO) that initiates the catabolism of tryptophan via the kynurenine pathway [12]. In addition, IFN-gamma stimulates the synthesis of neopterin by activated monocyte-derived macrophages and dendritic cells [14]. Previous epidemiological studies found KTR was associated with increased risk of lung cancer [15] or combined colorectal, breast, lung and prostate cancer [26]. Two studies examined the association between neopterin and cancer risk. One study found that both higher and lower levels of total neopterin (sum of neopterin and 7,8-dihydroneopterin) were associated with increased risk of colorectal cancer [16] whereas the other study found that only high level of neopterin was associated with increased risk of breast, colorectal, lung and prostate cancers combined [26]. The present study examined and did not find an association between KTR or neopterin and risk of pancreatic cancer. More studies are warranted to examine the role of KTR and neopterin in the development of pancreatic cancer.

The strengths of the current study include prospective design, long-term follow-up, and a comprehensive measurement of tryptophan and metabolites in the kynurenine pathway using a validated method of mass-spectrometry-based assay [24, 25]. In addition, we adjusted for potential confounders such as smoking, renal function (e.g., eGFR), and PLP, important determinants of levels of metabolites in the kynurenine pathway. Exclusion of first two or up to four years of pancreatic cancer cases did not materially change the inverse association between HAA or HAA:HK ratio and risk of pancreatic cancer, suggesting that the reverse causality was less likely. The present study had some limitations. The chief limitation of the present study was modest sample size although we utilized two large prospective cohort studies involving more than 80,000 participants with up to 20 years of follow-up. The small sample size may explain the null findings for some of the kyurenine metabolites with risk of pancreatic cancer. The measurement of biomarkers in blood samples at a single point of time with mixed fasting and nonfasting status may have resulted in measurement errors of the biomarkers. Such measurement errors most likely occurred equally for cases and matched controls in a non-differential fashion given the individually matched study design and the same batch of laboratory assay for both index case’s and the matched control’s samples. Such non-differential measurement errors usually lead to a null association. If it is true, we might underestimate the association between HAA or the HAA:HK ratio and the risk of pancreatic cancer in the present study. The lack of other established inflammatory biomarkers such as C-reactive protein may result in residual confounding on the observed association between HAA ratio and risk of pancreatic cancer. Future studies with a larger sample size and a more comprehensive measurement of inflammatory biomarkers are warranted to validate our findings.

In conclusion, the present study report inverse associations between HAA or the HAA:HK ratio and risk of pancreatic cancer. These results support our previous findings of inverse associations between dietary vitamin B_6_ intake, serum PLP, and risk of pancreatic cancer. These findings further indicate an important role of functional vitamin B_6_ status in the development of pancreatic cancer. In addition, the associations of HAA or the HAA:HK ratio with pancreatic cancer risk may reflect mechanisms other than vitamin B_6_ status, given that HAA and the HAA:HK ratio were associated with reduced risk of pancreatic cancer even after adjustment for serum concentration of PLP. Future studies are needed to elucidate biological mechanisms for tryptophan and kynurenine pathway in the development of pancreatic cancer.

## Supporting information

S1 Table Within-batch and between-batch coefficients of variations (CV) of tryptophan, kynurenine metabolites and neopterin among all control subjects of both Shanghai and Singapore cohorts combined (N = 362). (DOCX)

S2 Table Range of tryptophan,kynurenine metabolites and neopterin among all control subjects of both Shanghai and Singapore cohorts combined (N = 362) and the Shanghai (n = 258) and Singapore cohort (n = 104) separately. (DOCX)

S3 Table Spearman correlation coefficients of serum tryptophan, kynurenine metabolites and neopterin among all control subjects of both Shanghai and Singapore cohorts combined (n = 362). (DOCX)

S4 Table Associations between tertile levels of tryptophan, kynurenine metabolites and neopterin and risk of pancreatic cancer, the Shanghai Cohort Study. (DOCX)

S5 Table Associations between tertile levels of tryptophan,kynurenine metabolites and neopterin and risk of pancreatic cancer, the Singapore Chinese Health Study. (DOCX)

S6 Table Associations of tryptophan and kynurenines with pancreatic cancer risk among individuals stratified by PLP deficiency status in both the Shanghai and Singapore cohorts combined. (DOCX)

## Abbreviations

AHR: aryl hydrocarbon receptor
BMI: body mass index
CI: confidence interval
eGFR: estimated glomerular filtration rate
HAA: 3-hydroxyanthranilic acid
HK: 3-hydroxykynurenine
IDO: Indoleamine 2,3-dioxygenase
IFN: gamma, Interferon-gamma
IL-17: interleukin-17
KA: kynurenic acid
KAT: kynurenine amino transferase
KTR: kynurenine:tryptophan ratio
KYNU: kynureninase
LC-MS/MS: liquid chromatography-tandem mass spectrometry
OR: Odds ratio
PLP: Pyridoxal 5’-phosphate
XA: xanthurenic acid

## References

[1] Howlader N, Noone AM, Krapcho M, Miller D, Bishop K, Altekruse SF, et al SEER Cancer Statistics Review, 1975–2013, National Cancer Institute Bethesda, MD, http://seer.cancer.gov/csr/1975_2013/, based on November 2015 SEER data submission, posted to the SEER web site, April 2016.

[2] Blackford A, Parmigiani G, Kensler TW, Wolfgang C, Jones S, Zhang X, et al Genetic mutations associated with cigarette smoking in pancreatic cancer. Cancer research. 2009;69(8):3681–8. doi: 10.1158/0008-5472.CAN-09-0015 ; PubMed Central PMCID: PMCPMC2669837. 19351817 10.1158/0008-5472.CAN-09-0015 PMC2669837

[3] Stolzenberg-Solomon RZ, Schairer C, Moore S, Hollenbeck A, Silverman DT. Lifetime adiposity and risk of pancreatic cancer in the NIH-AARP Diet and Health Study cohort. Am J Clin Nutr. 2013;98(4):1057–65. doi: 10.3945/ajcn.113.058123 ; PubMed Central PMCID: PMCPMC3778860. 23985810 10.3945/ajcn.113.058123 PMC3778860

[4] Kobayashi T, Nishiumi S, Ikeda A, Yoshie T, Sakai A, Matsubara A, et al A novel serum metabolomics-based diagnostic approach to pancreatic cancer. Cancer Epidemiol Biomarkers Prev. 2013;22(4):571–9. doi: 10.1158/1055-9965.EPI-12-1033 . 23542803 10.1158/1055-9965.EPI-12-1033

[5] Zhang G, He P, Tan H, Budhu A, Gaedcke J, Ghadimi BM, et al Integration of metabolomics and transcriptomics revealed a fatty acid network exerting growth inhibitory effects in human pancreatic cancer. Clin Cancer Res. 2013;19(18):4983–93. doi: 10.1158/1078-0432.CCR-13-0209 ; PubMed Central PMCID: PMCPMC3778077. 23918603 10.1158/1078-0432.CCR-13-0209 PMC3778077

[6] Mayers JR, Wu C, Clish CB, Kraft P, Torrence ME, Fiske BP, et al Elevation of circulating branched-chain amino acids is an early event in human pancreatic adenocarcinoma development. Nat Med. 2014;20(10):1193–8. doi: 10.1038/nm.3686 ; PubMed Central PMCID: PMCPMC4191991. 25261994 10.1038/nm.3686 PMC4191991

[7] Hirata Y, Kobayashi T, Nishiumi S, Yamanaka K, Nakagawa T, Fujigaki S, et al Identification of highly sensitive biomarkers that can aid the early detection of pancreatic cancer using GC/MS/MS-based targeted metabolomics. Clin Chim Acta. 2017;468:98–104. doi: 10.1016/j.cca.2017.02.011 . 28215548 10.1016/j.cca.2017.02.011

[8] Choi SW, Friso S. Vitamins B6 and cancer. Sub-cellular biochemistry. 2012;56:247–64. doi: 10.1007/978-94-007-2199-9_13 . 22116703 10.1007/978-94-007-2199-9_13

[9] Huang JY, Butler LM, Wang R, Jin A, Koh WP, Yuan JM. Dietary Intake of One-Carbon Metabolism-Related Nutrients and Pancreatic Cancer Risk: The Singapore Chinese Health Study. Cancer Epidemiol Biomarkers Prev. 2016;25(2):417–24. doi: 10.1158/1055-9965.EPI-15-0594 ; PubMed Central PMCID: PMCPMC4767683. 26711329 10.1158/1055-9965.EPI-15-0594 PMC4767683

[10] Huang JY, Butler LM, Midttun O, Koh WP, Ueland PM, Wang R, et al Serum B6 vitamers (pyridoxal 5'-phosphate, pyridoxal, and 4-pyridoxic acid) and pancreatic cancer risk: two nested case-control studies in Asian populations. Cancer Causes Control. 2016;27(12):1447–56. doi: 10.1007/s10552-016-0822-6 . 27830400 10.1007/s10552-016-0822-6 PMC5161671

[11] Ulvik A, Theofylaktopoulou D, Midttun O, Nygard O, Eussen SJ, Ueland PM. Substrate product ratios of enzymes in the kynurenine pathway measured in plasma as indicators of functional vitamin B-6 status. Am J Clin Nutr. 2013;98(4):934–40. doi: 10.3945/ajcn.113.064998 . 24004893 10.3945/ajcn.113.064998

[12] Chen Y, Guillemin GJ. Kynurenine pathway metabolites in humans: disease and healthy States. International journal of tryptophan research: IJTR. 2009;2:1–19. ; PubMed Central PMCID: PMC3195227. 22084578 10.4137/ijtr.s2097 PMC3195227

[13] Ueland PM, McCann A, Midttun O, Ulvik A. Inflammation, vitamin B6 and related pathways. Mol Aspects Med. 2016 doi: 10.1016/j.mam.2016.08.001 . 27593095 10.1016/j.mam.2016.08.001

[14] Pingle SK, Tumane RG, Jawade AA. Neopterin: Biomarker of cell-mediated immunity and potent usage as biomarker in silicosis and other occupational diseases. Indian journal of occupational and environmental medicine. 2008;12(3):107–11. doi: 10.4103/0019-5278.44690 ; PubMed Central PMCID: PMC2796748. 20040967 10.4103/0019-5278.44690 PMC2796748

[15] Chuang SC, Fanidi A, Ueland PM, Relton C, Midttun O, Vollset SE, et al Circulating biomarkers of tryptophan and the kynurenine pathway and lung cancer risk. Cancer Epidemiol Biomarkers Prev. 2014;23(3):461–8. doi: 10.1158/1055-9965.EPI-13-0770 . 24357106 10.1158/1055-9965.EPI-13-0770

[16] Aleksandrova K, Chuang SC, Boeing H, Zuo H, Tell GS, Pischon T, et al A prospective study of the immune system activation biomarker neopterin and colorectal cancer risk. J Natl Cancer Inst. 2015;107(4). doi: 10.1093/jnci/djv010 ; PubMed Central PMCID: PMCPMC4402364. 25713165 10.1093/jnci/djv010 PMC4402364

[17] Zuo H, Ueland PM, Ulvik A, Eussen SJ, Vollset SE, Nygard O, et al Plasma Biomarkers of Inflammation, the Kynurenine Pathway, and Risks of All-Cause, Cancer, and Cardiovascular Disease Mortality: The Hordaland Health Study. Am J Epidemiol. 2016;183(4):249–58. doi: 10.1093/aje/kwv242 ; PubMed Central PMCID: PMCPMC4753283. 26823439 10.1093/aje/kwv242 PMC4753283

[18] Pichler R, Fritz J, Heidegger I, Steiner E, Culig Z, Klocker H, et al Predictive and prognostic role of serum neopterin and tryptophan breakdown in prostate cancer. Cancer science. 2017;108(4):663–70. doi: 10.1111/cas.13171 ; PubMed Central PMCID: PMCPMC5406598. 28107600 10.1111/cas.13171 PMC5406598

[19] Isci Bostanci E, Ugras Dikmen A, Girgin G, Gungor T, Baydar T, Nuri Danisman A. A New Diagnostic and Prognostic Marker in Endometrial Cancer: Neopterin. Int J Gynecol Cancer. 2017;27(4):754–8. doi: 10.1097/IGC.0000000000000952 . 28383326 10.1097/IGC.0000000000000952

[20] Yuan JM, Ross RK, Wang XL, Gao YT, Henderson BE, Yu MC. Morbidity and mortality in relation to cigarette smoking in Shanghai, China. A prospective male cohort study. JAMA. 1996;275(21):1646–50. . 8637137

[21] Yuan JM, Stram DO, Arakawa K, Lee HP, Yu MC. Dietary cryptoxanthin and reduced risk of lung cancer: the Singapore Chinese Health Study. Cancer Epidemiol Biomarkers Prev. 2003;12(9):890–8. . 14504200

[22] Parkin DM, Whelan SI, Ferlay J, Teppo L, Thomas D. Cancer Incidence in Five Continents. Volume VII 2002.

[23] Ross RK, Yuan JM, Yu MC, Wogan GN, Qian GS, Tu JT, et al Urinary aflatoxin biomarkers and risk of hepatocellular carcinoma. Lancet. 1992;339(8799):943–6. . 1348796 10.1016/0140-6736(92)91528-g

[24] da Silva VR, Rios-Avila L, Lamers Y, Ralat MA, Midttun O, Quinlivan EP, et al Metabolite profile analysis reveals functional effects of 28-day vitamin B-6 restriction on one-carbon metabolism and tryptophan catabolic pathways in healthy men and women. J Nutr. 2013;143(11):1719–27. doi: 10.3945/jn.113.180588 ; PubMed Central PMCID: PMC3796343. 23966327 10.3945/jn.113.180588 PMC3796343

[25] Midttun O, Hustad S, Ueland PM. Quantitative profiling of biomarkers related to B-vitamin status, tryptophan metabolism and inflammation in human plasma by liquid chromatography/tandem mass spectrometry. Rapid Commun Mass Spectrom. 2009;23(9):1371–9. doi: 10.1002/rcm.4013 . 19337982 10.1002/rcm.4013

[26] Zuo H, Tell GS, Vollset SE, Ueland PM, Nygard O, Midttun O, et al Interferon-gamma-induced inflammatory markers and the risk of cancer: the Hordaland Health Study. Cancer. 2014;120(21):3370–7. doi: 10.1002/cncr.28869 ; PubMed Central PMCID: PMC4283722. 24948355 10.1002/cncr.28869 PMC4283722

[27] Breslow N, Day N. Statistical methods in cancer research, vol.1: The analysis of case–control studies Lyon: IARC: IARC Scientific Pub No. 32.; 1980. 7216345

[28] Levey AS, Stevens LA, Schmid CH, Zhang YL, Castro AF 3rd, Feldman HI, et al A new equation to estimate glomerular filtration rate. Annals of internal medicine. 2009;150(9):604–12. ; PubMed Central PMCID: PMC2763564. 19414839 10.7326/0003-4819-150-9-200905050-00006 PMC2763564

[29] Ogasawara N, Hagino Y, Kotake Y. Kynurenine-transaminase, kynureninase and the increase of xanthurenic acid excretion. J Biochem. 1962;52:162–6. . 13939554 10.1093/oxfordjournals.jbchem.a127591

[30] Stolzenberg-Solomon RZ, Albanes D, Nieto FJ, Hartman TJ, Tangrea JA, Rautalahti M, et al Pancreatic cancer risk and nutrition-related methyl-group availability indicators in male smokers. J Natl Cancer Inst. 1999;91(6):535–41. . 10088624 10.1093/jnci/91.6.535

[31] Schernhammer E, Wolpin B, Rifai N, Cochrane B, Manson JA, Ma J, et al Plasma folate, vitamin B6, vitamin B12, and homocysteine and pancreatic cancer risk in four large cohorts. Cancer research. 2007;67(11):5553–60. doi: 10.1158/0008-5472.CAN-06-4463 . 17545639 10.1158/0008-5472.CAN-06-4463

[32] Chuang SC, Stolzenberg-Solomon R, Ueland PM, Vollset SE, Midttun O, Olsen A, et al A U-shaped relationship between plasma folate and pancreatic cancer risk in the European Prospective Investigation into Cancer and Nutrition. Eur J Cancer. 2011;47(12):1808–16. doi: 10.1016/j.ejca.2011.02.007 ; PubMed Central PMCID: PMCPMC3500543. 21411310 10.1016/j.ejca.2011.02.007 PMC3500543

[33] Desvignes L, Ernst JD. Interferon-gamma-responsive nonhematopoietic cells regulate the immune response to Mycobacterium tuberculosis. Immunity. 2009;31(6):974–85. doi: 10.1016/j.immuni.2009.10.007 ; PubMed Central PMCID: PMC2807991. 20064452 10.1016/j.immuni.2009.10.007 PMC2807991

[34] McAllister F, Bailey JM, Alsina J, Nirschl CJ, Sharma R, Fan H, et al Oncogenic Kras activates a hematopoietic-to-epithelial IL-17 signaling axis in preinvasive pancreatic neoplasia. Cancer cell. 2014;25(5):621–37. doi: 10.1016/j.ccr.2014.03.014 ; PubMed Central PMCID: PMC4072043. 24823639 10.1016/j.ccr.2014.03.014 PMC4072043

[35] Lowe MM, Mold JE, Kanwar B, Huang Y, Louie A, Pollastri MP, et al Identification of cinnabarinic acid as a novel endogenous aryl hydrocarbon receptor ligand that drives IL-22 production. PloS one. 2014;9(2):e87877 doi: 10.1371/journal.pone.0087877 ; PubMed Central PMCID: PMC3912126. 24498387 10.1371/journal.pone.0087877 PMC3912126

[36] Koliopanos A, Kleeff J, Xiao Y, Safe S, Zimmermann A, Buchler MW, et al Increased arylhydrocarbon receptor expression offers a potential therapeutic target for pancreatic cancer. Oncogene. 2002;21(39):6059–70. doi: 10.1038/sj.onc.1205633 . 12203118 10.1038/sj.onc.1205633

