# Supplementary material for: A prospective evaluation of serum kynurenine metabolites and risk of pancreatic cancer
Source: PLoS One. 2018 May 7;13(5):e0196465. doi: 10.1371/journal.pone.0196465 (PMC5937773; doi:10.1371/journal.pone.0196465)
Supplement: S1 Table — (DOCX) [file pone.0196465.s001.docx]

S1 Table. Within-batch and between-batch coefficients of variations (CV) of tryptophan, kynurenine metabolites and neopterin among all control subjects of both Shanghai and Singapore cohorts combined (N=362)

| Biomarkers^1^ | Within-batch CV, % |  | Between-batch CV, % |
| --- | --- | --- | --- |
| Tryptophan, µmol/L | 0.9 |  | 1.1 |
| Kynurenine, nmol/L | 1.4 |  | 4.4 |
| AA, nmol/L | 5.5 |  | 6.4 |
| KA, nmol/L | 5.0 |  | 5.9 |
| HK, nmol/L | 4.2 |  | 3.8 |
| XA, nmol/L | 5.5 |  | 14.7 |
| HAA, nmol/L | 4.7 |  | 10.2 |
| Neopterin, nmol/L | 5.4 |  | 7.3 |

^1^Abbreviations: AA, anthranilic acid; HAA, 3-hydroxyanthranilic acid; HK, 3-hydroxykynurenine; KA, kynurenic acid; XA, xanthurenic acid.
