# Supplementary material for: A prospective evaluation of serum kynurenine metabolites and risk of pancreatic cancer
Source: PLoS One. 2018 May 7;13(5):e0196465. doi: 10.1371/journal.pone.0196465 (PMC5937773; doi:10.1371/journal.pone.0196465)
Supplement: S2 Table — (DOCX) [file pone.0196465.s002.docx]

S2 Table. Range of tryptophan,kynurenine metabolites and neopterin among all control subjects of both Shanghai and Singapore cohorts combined (N=362) and the Shanghai (n = 258) and Singapore cohort (n = 104) separately

|  | Range | | | | | |
| --- | --- | --- | --- | --- | --- | --- |
| Biomarkers^1^ | T1 | |  | T2 |  | T3 |
| Tryptophan, µmol/L | <69.7 (<72.0; <65.4) | |  | 69.7-80.1 (72.1-81.5; 65.5-76.2) |  | >80.1 (>81.5; >76.2) |
| Kynurenine, µmol/L | <1.45 (<1.45; <1,46)^2^ | |  | 1.45-1.72 (1.45-1.70; 1.47-1.77) |  | >1.72 (>1,70; >1.77) |
| AA, nmol/L | <16.4 (<15.7; <19.0) | |  | 16.4-23.0 (15.7-21.6; 19.0-26.2) |  | >23.0 (>21.6; >26.2) |
| KA, nmol/L | <46.4 (<45.9; <50.0) | |  | 46.4-63.5 (45.9-62.4; 50.0-66.3) |  | >63.5 (>62.4; >66.3) |
| HK, nmol/L | <39.5 (<39.0; <40.8) | |  | 39.5-52.6 (39.0-52.0; 40.8-52.9) |  | >52.6 (>52.0; >52.9) |
| XA, nmol/L | <14.7 (<14.9; <14.6) | |  | 14.7-21.3 (14.9-21.3; 14.6-20.8) |  | >21.3 (>21.3; >20.8) |
| HAA, nmol/L | <33.2 (<31.9; <38.7) | |  | 33.2-45.0 (31.9-43.6; 38.7-48.3) |  | >45.0 (>43.6; >48.3) |
| KA:HK ratio | | <1.00 (<1.00; <1.00) |  | 1.00-1.40 (1.00-1.40; 1.00-1.40) |  | >1.40 (>1.40; >1.40) |
| XA:HK ratio | | <0.326 (<0.33; <0.32) |  | 0.326-0.451 (0.33-0.46; 0.32-0.43) |  | >0.451 (>0.33; >0.43) |
| HAA:HK ratio | | <0.739 (<0.70; <0.84) |  | 0.739-0.994 (0.70-0.97; 0.84-1.03) |  | >0.994 (>0.97; >1.03) |
| KTR (x 100) | | <1.93 (<1.90; <2.06) |  | 1.93-2.28 (1.90-2.18; 2.06-2.51) |  | >2.28 (>2.18; >2.51) |
| Neopterin, nmol/L | | <13.8 (<12.7; <21.5) |  | 13.8-19.2 (12.8-16.1; 21.6-29.0) |  | >19.2 (>16.1; >29.0) |

^1^Abbreviations: AA, anthranilic acid; HAA, 3-hydroxyanthranilic acid; HK, 3-hydroxykynurenine; KA, kynurenic acid; XA, xanthurenic acid

^2^ the figures in the parenthesis were cohort specific cut-off tertile values (Shanghai cohort; Singapore cohort).
