# Supplementary material for: A prospective evaluation of serum kynurenine metabolites and risk of pancreatic cancer
Source: PLoS One. 2018 May 7;13(5):e0196465. doi: 10.1371/journal.pone.0196465 (PMC5937773; doi:10.1371/journal.pone.0196465)
Supplement: S3 Table — (DOCX) [file pone.0196465.s003.docx]

S3 Table. Spearman correlation coefficients of serum tryptophan, kynurenine metabolites and neopterin among all control subjects of both Shanghai and Singapore cohorts combined (n=362)

| Biomarkers^1^ | Tryptophan | Kynurenine | AA | HK | KA | XA | HAA | KA:HK ratio | XA:HK ratio | HAA:HK ratio | KTR | Neopterin |
| --- | --- | --- | --- | --- | --- | --- | --- | --- | --- | --- | --- | --- |
| PLP | 0.05 | 0.02 | 0.16^2^ | -0.14^2^ | 0.14^2^ | 0.07 | 0.27^3^ | 0.26^3^ | 0.24^3^ | 0.39^3^ | -0.02 | 0.39^3^ |
| Tryptophan |  | 0.34^3^ | -0.02 | 0.12^2^ | 0.13^2^ | 0.34^3^ | 0.40^3^ | 0.02 | 0.27^3^ | 0.30^3^ | -0.45^3^ | -0.22^3^ |
| Kynurenine |  |  | 0.35^3^ | 0.54^3^ | 0.50^3^ | 0.39^3^ | 0.36^3^ | 0.09 | -0.01 | -0.10 | 0.63^3^ | 0.30^3^ |
| AA |  |  |  | 0.15^2^ | 0.31^3^ | 0.17^2^ | 0.13^2^ | 0.20^2^ | 0.07 | 0.003 | 0.36^3^ | 0.29^3^ |
| HK |  |  |  |  | 0.49^3^ | 0.60^3^ | 0.47^3^ | -0.30^3^ | -0.15^2^ | -0.34^3^ | 0.42^3^ | 0.25^3^ |
| KA |  |  |  |  |  | 0.68^3^ | 0.47^3^ | 0.64^3^ | 0.41^3^ | 0.07 | 0.39^3^ | 0.24^3^ |
| XA |  |  |  |  |  |  | 0.61^3^ | 0.24^3^ | 0.65^3^ | 0.12^2^ | 0.12^2^ | 0.09 |
| HAA |  |  |  |  |  |  |  | 0.10 | 0.32^3^ | 0.61^3^ | 0.02 | 0.22^3^ |
| KA:HK ratio |  |  |  |  |  |  |  |  | 0.61^3^ | 0.37^3^ | 0.06 | 0.05 |
| XA:HK ratio |  |  |  |  |  |  |  |  |  | 0.49^3^ | -0.21^3^ | -0.10 |
| HAA:HK ratio |  |  |  |  |  |  |  |  |  |  | -0.34^3^ | 0.03 |
| KTR |  |  |  |  |  |  |  |  |  |  |  | 0.46^3^ |

^1^Abbreviations: AA, anthranilic acid; HAA, 3-hydroxyanthranilic acid; HK, 3-hydroxykynurenine; KA, kynurenic acid; Kyn, kynurenine; PLP, pyridoxal 5’-phosphate; XA, xanthurenic acid

^2^P < 0.05, ^3^P < 0.0001
