# Supplementary material for: A prospective evaluation of serum kynurenine metabolites and risk of pancreatic cancer
Source: PLoS One. 2018 May 7;13(5):e0196465. doi: 10.1371/journal.pone.0196465 (PMC5937773; doi:10.1371/journal.pone.0196465)
Supplement: S4 Table — (DOCX) [file pone.0196465.s004.docx]

S4 Table. Associations between tertile levels of tryptophan, kynurenine metabolites and neopterin and risk of pancreatic cancer, The Shanghai Cohort Study

| Biomarkers^1^ | T1 | |  | T2 | |  | T3 | | P trend |
| --- | --- | --- | --- | --- | --- | --- | --- | --- | --- |
|  | Co/Ca^2^ | OR^3^ (ref) |  | Co/Ca^2^ | OR (95%CI)^3^ |  | Co/Ca^2^ | OR (95%CI)^3^ |  |
| Tryptophan | 66/40 | 1.00 |  | 94/42 | 0.66 (0.37-1.15) |  | 98/47 | 0.71 (0.41-1.24) | 0.24 |
| Kynurenine | 87/55 | 1.00 |  | 92/42 | 0.76 (0.44-1.31) |  | 79/32 | 0.65 (0.35-1.19) | 0.15 |
| AA | 97/39 | 1.00 |  | 80/49 | 2.25 (1.23-4.09) |  | 72/37 | 1.63 (0.86-3.11) | 0.13 |
| KA | 89/50 | 1.00 |  | 92/42 | 0.93 (0.53-1.63) |  | 77/37 | 0.90 (0.51-1.59) | 0.71 |
| HK | 88/46 | 1.00 |  | 80/42 | 1.06 (0.61-1.85) |  | 81/37 | 0.77 (0.43-1.36) | 0.37 |
| XA | 83/44 | 1.00 |  | 88/41 | 1.03 (0.6-1.78) |  | 87/44 | 1.01 (0.59-1.75) | 0.96 |
| HAA | 95/57 | 1.00 |  | 81/33 | 0.64 (0.36-1.12) |  | 73/35 | 0.76 (0.43-1.33) | 0.25 |
| KA:HK ratio | 82/50 | 1.00 |  | 85/38 | 0.71 (0.38-1.30) |  | 82/37 | 0.78 (0.43-1.42) | 0.46 |
| XA:HK ratio | 78/50 | 1.00 |  | 84/28 | 0.50 (0.26-0.93) |  | 87/47 | 0.84 (0.48-1.48) | 0.62 |
| HAA:HK ratio | 95/61 | 1.00 |  | 80/35 | 0.64 (0.37-1.09) |  | 74/29 | 0.58 (0.32-1.04) | 0.053 |
| KTR | 94/51 | 1.00 |  | 98/51 | 0.97 (0.55-1.72) |  | 66/27 | 0.89 (0.46-1.75) | 0.75 |
| Neopterin^4^ | 83/57 | 1.00 |  | 85/34 | 0.64 (0.35-1.17) |  | 81/34 | 0.71 (0.38-1.33) | 0.28 |

^1^Abbreviations: AA, anthranilic acid; HAA, 3-hydroxyanthranilic acid; HK, 3-hydroxykynurenine; KA, kynurenic acid; XA, xanthurenic acid.

^2^Co/Ca: number of control subjects/number of lung cancer cases.

^3^Odds ratios (ORs) and 95% confidence intervals (CIs) were derived from conditional logistic regression models that also included following covariates: education (no schooling, primary school, secondary school and higher), body mass index (<18.5, 18.5-<23.0, ≥23.0 kg/m^2^), smoking status (never, former, current smokers), serum cotinine concentrations (tertiles), alcohol drinking (drinks of alcoholic beverages per week), diabetes status (no, yes), concentration of pyridoxal 5’-phosphate (nmol/L) , and estimated glomerular filtration rate (mL/min/1.73m^2^).

^4^Cohort-specific tertiles was used for neopterin due to its different distribution between the Shanghai and Singapore cohorts.
