# Supplementary material for: A prospective evaluation of serum kynurenine metabolites and risk of pancreatic cancer
Source: PLoS One. 2018 May 7;13(5):e0196465. doi: 10.1371/journal.pone.0196465 (PMC5937773; doi:10.1371/journal.pone.0196465)
Supplement: S5 Table — (DOCX) [file pone.0196465.s005.docx]

S5 Table. Associations between tertile levels of tryptophan,kynurenine metabolites and neopterin and risk of pancreatic cancer, The Singapore Chinese Health Study

| Biomarkers^1^ | T1 |  |  | T2 |  |  | T3 |  | P trend | P^4^ |
| --- | --- | --- | --- | --- | --- | --- | --- | --- | --- | --- |
|  | Co/Ca^2^ | OR^3^ (ref) |  | Co/Ca^2^ | OR (95%CI)^3^ |  | Co/Ca^2^ | OR (95%CI)^3^ |  |  |
| Tryptophan | 54/26 | 1.00 |  | 29/21 | 1.58 (0.69-3.61) |  | 21/11 | 1.17 (0.43-3.15) | 0.63 | 0.49 |
| Kynurenine | 33/21 | 1.00 |  | 31/14 | 0.65 (0.26-1.59) |  | 40/23 | 1.12 (0.39-3.24) | 0.89 | 0.73 |
| AA | 20/8 | 1.00 |  | 40/28 | 1.81 (0.64-5.12) |  | 44/22 | 1.21 (0.37-3.97) | 0.90 | 0.35 |
| KA | 31/19 | 1.00 |  | 31/22 | 1.18 (0.48-2.91) |  | 42/17 | 0.66 (0.24-1.79) | 0.42 | 0.60 |
| HK | 31/22 | 1.00 |  | 38/20 | 0.62 (0.26-1.44) |  | 35/16 | 0.41 (0.14-1.18) | 0.09 | 0.36 |
| XA | 37/29 | 1.00 |  | 36/17 | 0.55 (0.24-1.22) |  | 31/12 | 0.39 (0.14-1.11) | 0.05 | 0.13 |
| HAA | 22/22 | 1.00 |  | 39/19 | 0.44 (0.18-1.08) |  | 43/17 | 0.34 (0.13-0.92) | 0.03 | 0.10 |
| KA:HK ratio | 35/14 | 1.00 |  | 35/29 | 2.43 (0.99-5.95) |  | 34/15 | 1.41 (0.52-3.8) | 0.49 | 0.31 |
| XA:HK ratio | 39/27 | 1.00 |  | 36/16 | 0.76 (0.31-1.87) |  | 29/15 | 0.84 (0.32-2.17) | 0.71 | 0.74 |
| HAA:HK ratio | 22/16 | 1.00 |  | 40/23 | 0.64 (0.23-1.79) |  | 42/19 | 0.54 (0.17-1.74) | 0.32 | 0.97 |
| KTR | 26/14 | 1.00 |  | 25/16 | 1.17 (0.42-3.29) |  | 53/28 | 1.07 (0.41-2.78) | 0.94 | 0.82 |
| Neopterin^5^ | 35/19 | 1.00 |  | 35/16 | 0.90 (0.38-2.11) |  | 34/23 | 1.55 (0.62-3.84) | 0.37 | 0.11 |

^1^Abbreviations: AA, anthranilic acid; HAA, 3-hydroxyanthranilic acid; HK, 3-hydroxykynurenine; KA, kynurenic acid; XA, xanthurenic acid.

^2^ Co/Ca: number of control subjects/number of lung cancer cases.

^3^Odds ratios (ORs) and 95% confidence intervals (CIs) were derived from conditional logistic regression models that also included following covariates: education (no schooling, primary school, secondary school and higher), body mass index (<18.5, 18.5-<23.0, ≥23.0 kg/m^2^), smoking status (never, former, current smokers), serum cotinine concentrations (tertiles), alcohol drinking (drinks of alcoholic beverages per week), diabetes status (no, yes), concentration of pyridoxal 5’-phosphate (nmol/L), and estimated glomerular filtration rate (mL/min/1.73m^2^).

^4^P values for the interaction between biomarker and cohort on risk of pancreatic cancer.

^5^Cohort-specific tertiles was used for neopterin due to its different distribution between the Shanghai and Singapore cohorts.
