# Supplementary material for: A prospective evaluation of serum kynurenine metabolites and risk of pancreatic cancer
Source: PLoS One. 2018 May 7;13(5):e0196465. doi: 10.1371/journal.pone.0196465 (PMC5937773; doi:10.1371/journal.pone.0196465)
Supplement: S6 Table — (DOCX) [file pone.0196465.s006.docx]

S6 Table. Associations of tryptophan and kynurenines with pancreatic cancer risk among individuals stratified by PLP deficiency status in both the Shanghai and Singapore cohorts combined

|  | PLP deficiency (< 20 nmol/L) | | | |  | | PLP sufficiency (≥ 20 nmol/L) | | | | P-interaction |  |
| --- | --- | --- | --- | --- | --- | --- | --- | --- | --- | --- | --- | --- |
|  | Co/Ca | | OR(95%CI) ^2^ | |  | | Co/Ca | OR(95%CI) ^2^ | | |  |  |
| Tryptophan |  | |  | |  | |  |  | | | 0.44 |  |
| 1^st^ tertile | 37/21 | | 1.00 (Ref) | |  | | 83/45 | 1.00 (Ref) | | |  |  |
| 2^nd^ tertile | 30/22 | | 1.02 (0.43-2.38) | |  | | 93/41 | 0.80 (0.46-1.39) | | |  |  |
| 3^rd^ tertile | 22/15 | | 1.05 (0.41-2.66) | |  | | 97/43 | 0.86 (0.49-1.52) | | |  |  |
| P-trend |  | | 0.92 | |  | |  | 0.62 | | |  |  |
| Kynurenine |  | |  | |  | |  |  | | | 0.21 |  |
| 1^st^ tertile | 26/25 | | 1.00 (Ref) | |  | | 94/51 | 1.00 (Ref) | | |  |  |
| 2^nd^ tertile | 34/22 | | 0.64 (0.27-1.54) | |  | | 89/34 | 0.65 (0.37-1.13) | | |  |  |
| 3^rd^ tertile | 29/11 | | 0.29 (0.1-0.85) | |  | | 90/44 | 0.88 (0.48-1.60) | | |  |  |
| P-trend |  | | 0.03 | |  | |  | 0.59 | | |  |  |
| Anthranilic acid |  | |  | |  | |  |  | | | 0.16 |  |
| 1^st^ tertile | 32/22 | | 1.00 (Ref) | |  | | 85/25 | 1.00 (Ref) | | |  |  |
| 2^nd^ tertile | 25/18 | | 1.23 (0.5-3.01) | |  | | 95/59 | 2.25 (1.25-4.02) | | |  |  |
| 3^rd^ tertile | 26/15 | | 1.00 (0.39-2.52) | |  | | 90/44 | 1.76 (0.93-3.33) | | |  |  |
| P-trend |  | | 0.97 | |  | |  | 0.12 | | |  |  |
| KA |  | |  | |  | |  |  | | | 0.06 |  |
| 1^st^ tertile | 32/25 | | 1.00 (Ref) | |  | | 88/44 | 1.00 (Ref) | | |  |  |
| 2^nd^ tertile | 33/22 | | 0.87 (0.37-2.02) | |  | | 90/42 | 1.00 (0.56-1.76) | | |  |  |
| 3^rd^ tertile | 24/11 | | 0.42 (0.15-1.17) | |  | | 95/43 | 1.02 (0.57-1.83) | | |  |  |
| P-trend |  | | 0.12 | |  | |  | 0.94 | | |  |  |
| HK |  | |  | |  | |  |  | | | 0.07 |  |
| 1^st^ tertile | 24/22 | | 1.00 (Ref) | |  | | 95/46 | 1.00 (Ref) | | |  |  |
| 2^nd^ tertile | 21/18 | | 0.75 (0.29-1.90) | |  | | 97/44 | 0.95 (0.56-1.62) | | |  |  |
| 3^rd^ tertile | 38/15 | | 0.31 (0.12-0.84) | |  | | 78/38 | 1.03 (0.57-1.85) | | |  |  |
| P-trend |  | | 0.02 | |  | |  | 0.94 | | |  |  |
| XA | |  |  |  | |  | | |  | 0.11 | | |
| 1^st^ tertile | | 34/26 | 1.00 (Ref) |  | | 86/47 | | | 1.00 (Ref) |  | | |
| 2^nd^ tertile | | 24/20 | 1.17 (0.47-2.88) |  | | 100/38 | | | 0.78 (0.45-1.35) |  | | |
| 3^rd^ tertile | | 31/12 | 0.37 (0.14-0.99) |  | | 87/44 | | | 1.14 (0.65-2.01) |  | | |
| P-trend | |  | 0.07 |  | |  | | | 0.66 |  | | |
| HAA | |  |  |  | |  | | |  | 0.46 | | |
| 1^st^ tertile | | 39/33 | 1.00(ref) |  | | 78/46 | | | 1.00 (Ref) |  | | |
| 2^nd^ tertile | | 27/15 | 0.35 (0.14-0.90) |  | | 93/37 | | | 0.66 (0.37-1.15) |  | | |
| 3^rd^ tertile | | 17/7 | 0.40 (0.13-1.16) |  | | 99/45 | | | 0.80 (0.46-1.39) |  | | |
| P-trend | |  | 0.04 |  | |  | | | 0.45 |  | | |
| KA:HK ratio | |  |  |  | |  | | |  | 0.30 | | |
| 1^st^ tertile | | 38/23 | 1.00 (Ref) |  | | 79/41 | | | 1.00 (Ref) |  | | |
| 2^nd^ tertile | | 22/22 | 1.52 (0.62-3.74) |  | | 98/45 | | | 0.99 (0.57-1.7) |  | | |
| 3^rd^ tertile | | 23/10 | 0.63 (0.23-1.73) |  | | 93/42 | | | 1.00 (0.57-1.74) |  | | |
| P-trend | |  | 0.49 |  | |  | | | 0.99 |  | | |
| XA:HK ratio | |  |  |  | |  | | |  | 0.72 | | |
| 1^st^ tertile | | 41/29 | 1.00 (Ref) |  | | 76/48 | | | 1.00 (Ref) |  | | |
| 2^nd^ tertile | | 23/13 | 0.46 (0.17-1.24) |  | | 97/31 | | | 0.57 (0.32-1.01) |  | | |
| 3^rd^ tertile | | 19/13 | 0.67 (0.26-1.74) |  | | 97/49 | | | 0.98 (0.57-1.66) |  | | |
| P-trend | |  | 0.33 |  | |  | | | 0.98 |  | | |
| HAA:HK ratio | |  |  |  | |  | | |  | 0.09 | | |
| 1^st^ tertile | | 45/35 | 1.00 (Ref) |  | | 72/42 | | | 1.00 (Ref) |  | | |
| 2^nd^ tertile | | 24/15 | 0.71 (0.3-1.68) |  | | 96/43 | | | 0.74 (0.42-1.29) |  | | |
| 3^rd^ tertile | | 14/5 | 0.29 (0.09-0.98) |  | | 102/43 | | | 0.74 (0.42-1.3) |  | | |
| P-trend | |  | 0.0496 |  | |  | | | 0.32 |  | | |

Abbreviations: HAA, 3-hydroxyanthranilic acid; HK, 3-hydroxykynurenine; KA, kynurenic acid; XA, xanthurenic acid

^2^ Odds ratios were derived from unconditional logistic regression models that also included following covariates: education (no schooling, primary school, secondary school and higher), body mass index (<18.5, 18.5-<23.0, ≥23.0), smoking status (never, former, current smokers), serum cotinine (nmol/L), alcohol drinking (drinkers of alcoholic beverages per week), diabetes status (no, yes), and estimated glomerular filtration rate (mL/min/1.73 m^2^)
